# Fixing the Leaky Pipe: How to Improve the Uptake of Patient-Reported Outcomes–Based Prognostic and Predictive Models in Cancer Clinical Practice

**DOI:** 10.1200/CCI.23.00070

**Published:** 2023-11-17

**Authors:** Katie L. Spencer, Kate L. Absolom, Matthew J. Allsop, Samuel D. Relton, Jessica Pearce, Kuan Liao, Sairah Naseer, Omolola Salako, Daniel Howdon, Jenny Hewison, Galina Velikova, Corinne Faivre-Finn, Hilary L. Bekker, Sabine N. van der Veer

**Affiliations:** ^1^ Leeds Institute of Health Sciences, University of Leeds, Leeds, United Kingdom; ^2^ Leeds Cancer Centre, Leeds Teaching Hospitals NHS Trust, Leeds, United Kingdom; ^3^ Leeds Institute of Data Analytics, University of Leeds, Leeds, United Kingdom; ^4^ Leeds Institute of Medical Research, University of Leeds, Leeds, United Kingdom; ^5^ Division of Informatics, Imaging and Data Sciences, Faculty of Biology, Medicine and Health, Centre for Health Informatics, Manchester Academic Health Science Centre, The University of Manchester, Manchester, United Kingdom; ^6^ School of Medicine, University of Leeds, Leeds, United Kingdom; ^7^ College of Medicine, University of Lagos, Lagos, Nigeria; ^8^ Institute of Cancer Sciences, University of Manchester, Manchester, United Kingdom

## Abstract

**PURPOSE:**

This discussion paper outlines challenges and proposes solutions for successfully implementing prediction models that incorporate patient-reported outcomes (PROs) in cancer practice.

**METHODS:**

We organized a full-day multidisciplinary meeting of people with expertise in cancer care delivery, PRO collection, PRO use in prediction modeling, computing, implementation, and decision science. The discussions presented here focused on identifying challenges to the development, implementation and use of prediction models incorporating PROs, and suggesting possible solutions.

**RESULTS:**

Specific challenges and solutions were identified across three broad areas. (1) Understanding decision making and implementation: necessitating multidisciplinary collaboration in the early stages and throughout; early stakeholder engagement to define the decision problem and ensure acceptability of PROs in prediction; understanding patient/clinician interpretation of PRO predictions and uncertainty to optimize prediction impact; striving for model integration into existing electronic health records; and early regulatory alignment. (2) Recognizing the limitations to PRO collection and their impact on prediction: incorporating validated, clinically important PROs to maximize model generalizability and clinical engagement; and minimizing missing PRO data (resulting from both structural digital exclusion and time-varying factors) to avoid exacerbating existing inequalities. (3) Statistical and modeling challenges: incorporating statistical methods to address missing data; ensuring predictive modeling recognizes complex causal relationships; and considering temporal and geographic recalibration so that model predictions reflect the relevant population.

**CONCLUSION:**

Developing and implementing PRO-based prediction models in cancer care requires extensive multidisciplinary working from the earliest stages, recognition of implementation challenges because of PRO collection and model presentation, and robust statistical methods to manage missing data, causality, and calibration. Prediction models incorporating PROs should be viewed as complex interventions, with their development and impact assessment carried out to reflect this.

## Teaser

Challenges and solutions to implementing patient-reported outcome–based predictive models in cancer.

## INTRODUCTION

Cancer care and research have progressed rapidly over recent decades. Restricted patient cohorts in clinical trials of new treatments, however, limit the direct relevance of trial results in routine care settings.^1^ Partly in response to this, studies now aim to develop models capable of predicting outcomes (eg, survival or symptom trajectories) to support clinicians and patients in making shared and personalized decisions in routine cancer care.^2,3^ Given the documented optimism of clinicians delivering cancer care, such predictions may help patients and clinicians make more informed treatment decisions that align with patient preferences, reduce the likelihood of decisions to pursue overly aggressive cancer care in the final months of life, inform trial participation, when and what to tell family members, and how to prepare for the end of life.^4-10^

Models are developed in curative and adjuvant,^11,12^ as well as advanced incurable cancer settings.^13-15^ The models effectively identify a weighting for observed characteristics to provide predictions of expected outcomes for individuals. These models, however, rarely make it into clinical practice, meaning opportunities to better inform treatment decisions are missed.^2,16,17^

Models predicting outcomes may be prognostic (indicating expected prognosis or outcomes) or predictive (indicating expected outcomes conditional upon a given treatment approach). Here, we will use the term prediction models to encompass both modeling approaches.

### Models in Cancer Care

Typically, prediction models in cancer care include a range of clinical parameters, such as clinical test results, patient or tumor characteristics, biomarkers, and performance status (ie, physician-assessed measurement of function).^13,18-21^ Performance status is a key parameter in many such models but was originally developed to inform patient selection for clinical trials and, although never formally validated beyond this, is now widely used to assess suitability for systemic anticancer treatment.^22^ Its subjectivity and lack of granularity are increasingly recognized limitations for its ability to assess patients' functioning and suitability for treatment,^22-24^ leading to a call for incorporating more comprehensive measures of functioning and frailty into treatment decisions, particularly in the context of an aging population.^22,25^ Indeed, patient-reported outcome (PRO) measures are now available to support this.^26-28^ For example, both the Geriatric-8 and Vulnerable Elders Survey-13 offer patient-reported questionnaire–based tools that assess frailty on the basis of multiple domains, including function/mobility, nutrition, and polypharmacy.^26,29^

### PROs in Prediction Models

**FIG 1.**
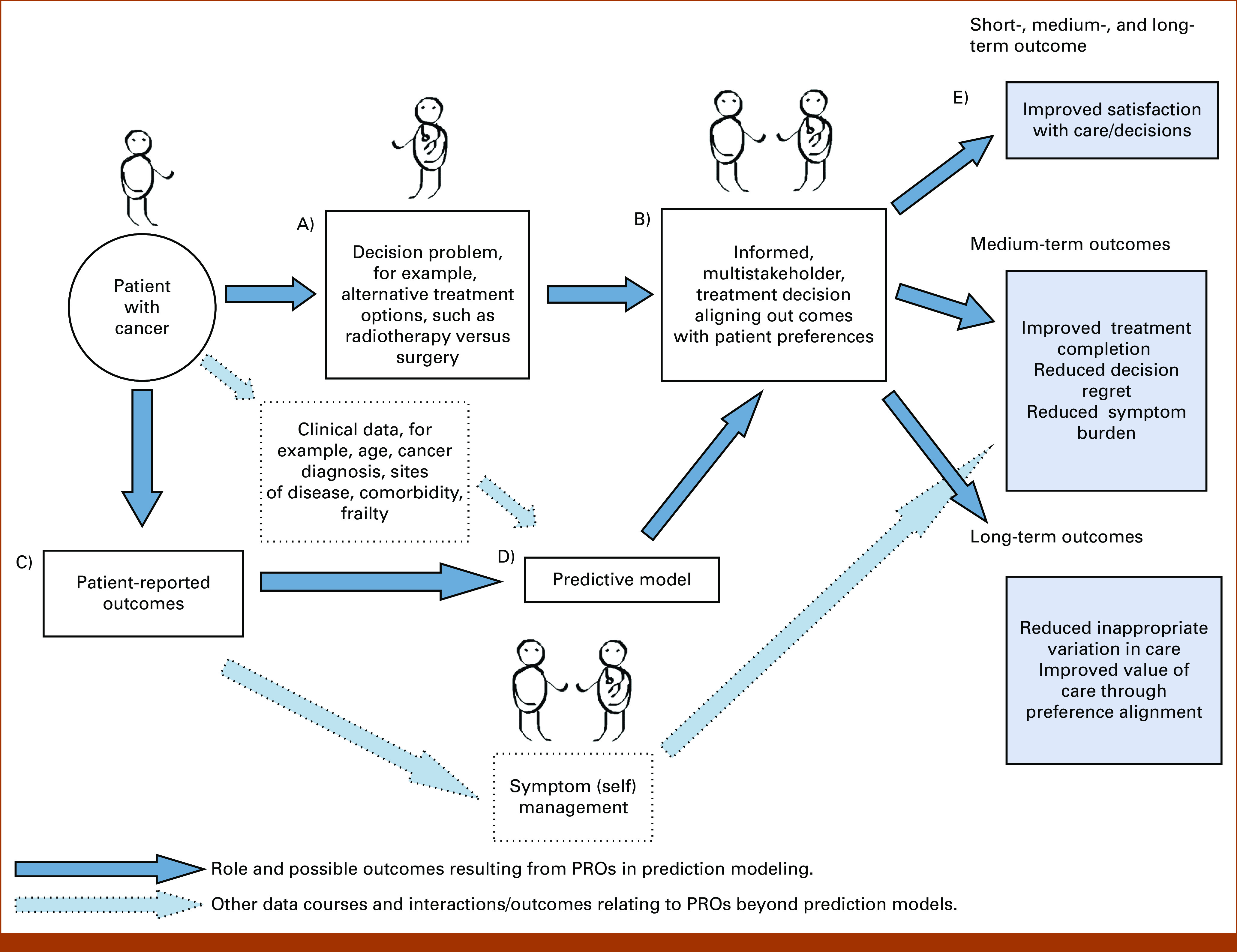
Conceptual framework for the role of PROs in predictive modeling. PROs, patient-reported outcomes.

There is wide consensus that PROs can enhance routine cancer care by streamlining consultations to focus on areas that are important to patients, thus aiding symptom control, empowering patients, reducing health service utilization, and, in some circumstances, improving overall survival and quality of life (QOL).^30-36^ Furthermore, PROs have prognostic value,^37-40^ while also providing a patient-reported analog for specialist clinician assessment of symptoms.^30^ This makes PROs suitable for capturing clinically important information for prediction modeling ahead of or in the absence of specialist review. Finally, when captured repeatedly over time in specialist settings, they may also replace variables such as performance status in prediction models.^41^ Together, this suggests that incorporating PROs into prediction models for cancer care should be explored further. A conceptual framework for the role of such models is presented in Figure 1.

### Improving the Uptake of PRO-Based Prediction Models—A Multidisciplinary Workshop

**FIG 2.**
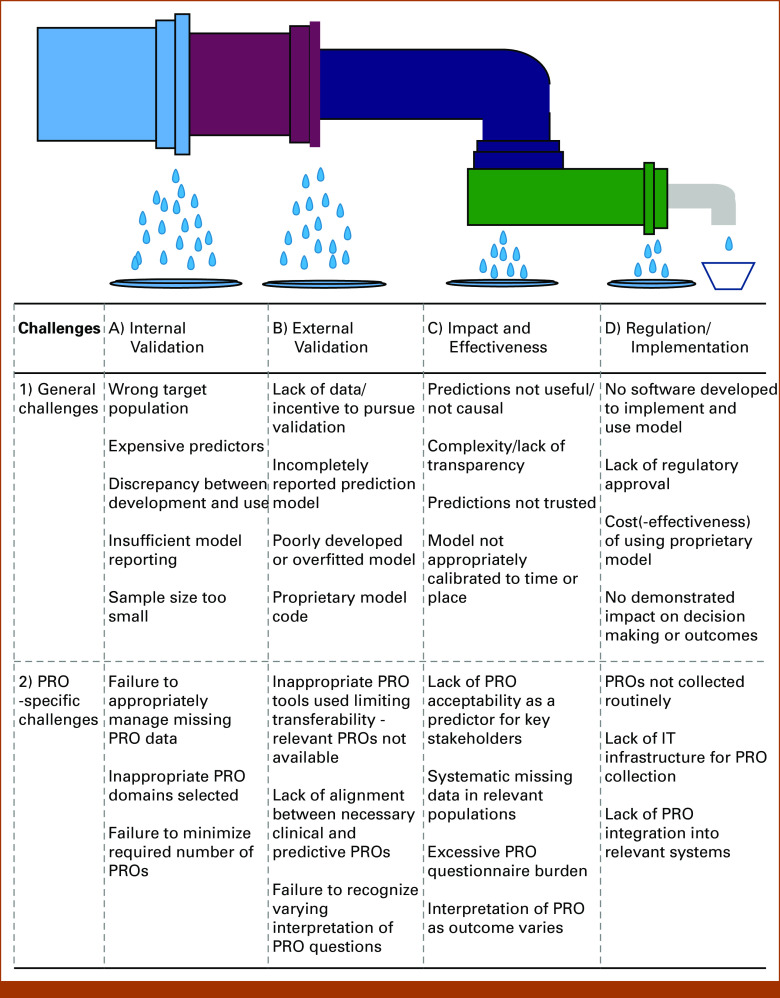
The leaky pipe—why developed prognostic and predictive models do not make it into clinical practice. IT, information technology; PRO, patient-reported outcome. Adapted with permission from Royen et al.^43^

We conducted a multidisciplinary workshop to further explore and reach consensus on the challenges and approaches for research into harnessing PROs for prediction modeling in cancer care. This perspective paper reports on the outcomes of the workshop, considering the whole (often leaky^42,43^) pipeline from identifying the decision problem through to successful implementation in clinical practice (Fig 2), with specific focus on how these challenges relate to the use of PROs.

The workshop identified the crucial need, applicable to any prediction model pipeline, to start by carefully defining the decision problem, that is, the clinical decision the model aims to influence (Fig 1A). Following immediately from this is the need to identify key stakeholders and consider the pathway to implementation.^17^ As such, we start our summary with key aspects of model implementation, given the crucial role this has in underpinning all subsequent progress. We then move on to considering how the capture of PROs in routine care will affect model development and highlight some key statistical considerations. In doing this, we aim to suggest next steps by which the leaky pipeline may be fixed. Table 1 displays a summary of the points raised.

**TABLE 1.**
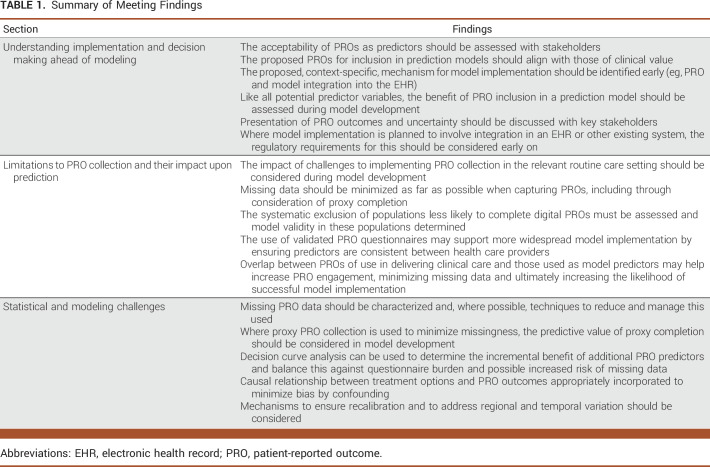
Summary of Meeting Findings

## METHODS

Two authors (K.L.S. and H.L.B.) developed an outline for a full-day meeting (7 hours) to cover key areas as outlined by the PROGRESS reports (the PROGnosis RESearch Strategy series, which seeks to explain how prognostic modeling research can be used to improve clinical outcomes).^17^ Relevant multidisciplinary attendees were identified from the author's existing networks, snowballing from these to ensure program coverage. Attendees were invited by email.

Twenty-three people attended, including those with expertise in routine cancer care delivery (n = 6); the use of PROs in cancer care (n = 4); large-scale PRO data analysis (n = 6) and prediction modeling (n = 5); health informatics (n = 4) and computing (n = 4); implementation (n = 7); and decision science (n = 1). Some attendees had expertise in more than one area. The first author (K.L.S.) facilitated the meeting. Expert presentations included the challenges of PRO implementation in cancer care; PRO-based analyses in cancer care (including prognostic modeling); the challenges of translating model predictions into clinical practice from a clinician perspective and separately from an informatics and implementation science perspective; and how model predictions may inform decision making. Each presentation was followed by an extended opportunity for discussion. Key points were documented during the meeting and subsequently thematically analyzed and synthesized with multiple rounds of review by all coauthors.

Discussions focused principally on the high-resource setting, extending to low-resource settings, where possible, if expertise was available within the group.

### Starting With the Last Drop—Understanding Implementation and Decision Making Ahead of Modeling

#### Defining the Decision Problem

An insufficient understanding of the decision problem and the intended role of the prediction model in this will hamper model implementation (Figs 1A, 1D and 2). Decision scientists have provided evidence and theories to understand how people make decisions under (un)certainty and risk.^44^ The theoretical approaches are informed by (1) economic and statistical modeling of the ideal decision,^45^ (2) descriptions of how people make decisions naturalistically,^46^ and (3) evidence to inform active thinking in real-world contexts.^47^ Broadly, these models identify the components on which people draw to make decisions such as the options (eg, available treatments), attributes (eg, treatment practicalities and side effects), probabilities (eg, expected survival and side-effect probabilities), and values individuals place on these consequences. Decision science has identified the types of factors that affect our attention and help or hinder our decision-making processes. Furthermore, in health care, most decisions are made by multiple stakeholders with different goals (Fig 1B).^48^ Actions arising from multiple stakeholder decision making require a shared understanding of other people's perspectives to agree and implement a health care choice of benefit and relevance to the patient.^49^ Prediction models could contribute to interventions designed to support clinical reasoning and shared decision making between multiple stakeholders.

When developing a prediction model, working within a multidisciplinary team from the earliest possible stage, there is a need to use decision science theories and all available evidence to pinpoint the specific clinical decision (Fig 1A); determine the aims of providing model predictions (eg, to communicate risk, inform patients/clinicians, or reduce variation; Fig 1E); and identify the individual/s who will interact with these (Fig 1D). On the basis of this information, it is then critical to define which outcomes (and time horizons) will be of most value; determining the key outcomes of interest at an early stage can ensure that model outcomes will be actionable (Figs 1D and 2).

#### Models as Complex Interventions

The greater regulation of prediction models as medical devices and increasing desire to use them in routine care highlight the need to assess model impact ahead of widespread implementation.^17,50^ The complexity of the decisions and environments in which these models are implemented warrants consideration of the updated Medical Research Council guidance on developing and evaluating complex interventions.^51^ A program theory should describe assumptions on how the model is expected to deliver the desired outcomes and how different individuals and features of the context may interact with the final model to influence the success of implementation^51^ (eg, on then basis of Fig 1). These assumptions may need revisiting and refinement during exploratory and preparatory phases (considering feasibility) with input from relevant stakeholders (eg, patients, clinicians, and information technology teams) ahead of impact assessment.^50,51^ For example, is it realistic to think that predictions will be acted upon, even if they conflict with hoped-for outcomes or interventions? Do all stakeholders accept the role of PROs as predictors or outcomes (Figs 1C and 2C)? Furthermore, clinicians may be concerned about issues of overdependence on models, loss of clinical autonomy, and lack of confidence in the model predictions (Figs 1B, 1C and 2).^52^ These concerns/considerations should be documented and addressed (eg, through training for clinicians and parallel patient information) to support wider implementation and dissemination.

#### Enabling Implementation

A range of factors should be considered when planning implementation. Model predictions must be presented in a timely way to influence clinical care (Figs 1C, 1D and 2). With PRO prediction models, wider integration of PROs into electronic health records (EHRs) may enable this (Fig 2D)^53-55^ and avoid the presentation of predictions after the decision point. This also minimizes the need for multiple logins or opening a separate webpage to access predictions, both of which are recognized barriers to implementing routine PRO collection and model-based interventions.^52,56,57^ When integrated, the risk of repeated alerts interrupting clinical workflows and leading to alert fatigue may also be a potential barrier to model implementation.^56,57^ Conversely, the presentation of predictions should not introduce attention bias, as the decision-maker allocates greater importance to the model outputs than other relevant domains that inform the decision-making process (Fig 1B).

Finally, prediction models are increasingly used in low-resource cancer care settings^58,59^ because of wider EHR system implementation alongside increasing recognition of the role and value of PROs.^60,61^ PROs can be readily delivered with the support of mobile devices to facilitate patient-centered care in geographically dispersed populations and inform health service responses, for example, in patients nearing the end of life.^61,62^ Context-specific model implementation strategies are, however, required. For example, they must be congruent with clinical workflows and a limited health workforce,^63,64^ recognize the high frequency of advanced disease in low-resource settings,^65^ account for treatment availability and financial toxicity (Figs 1C and 2), adapt to unreliable internet connections and electricity supply, and support a business case for investment in infrastructure to enable access to timely, reliable, and practical health information, a prerequisite for delivering universal health coverage.^66^

#### Presenting Model Outcomes

Once the mechanism for presenting model predictions has been defined, the presentation needs tailoring to the decision-makers’ needs (Fig 2C). Prediction models that are directive rather than assistive when implemented (ie, not only present an expected outcome but also a decision recommendation) may be more readily incorporated into clinical practice.^52^ This may be challenging, however, where predictions of PRO domains are included, as the relative value placed on domains may differ between patients (whether due to language, culture, or personal preference; Fig 2B). It may also be necessary to provide details of the model inputs (eg, PRO dimensions) and rationale behind the recommendation (Figs 1C and 2).^52^ Although, patients may value model accuracy more than explainability for health care decision making, and are therefore willing to accept the black-box nature of models,^67^ clinicians may be more willing to use model predictions if clinical utility and validity has been demonstrated (Figs 1D and 2).^52^

The presentation of statistical uncertainty should also be considered during model development; for example, how should survival and patient-reported QOL, and the uncertainty around it, be presented, especially if communicated to patients? Presentation and visualization is particularly important when considering longitudinal PRO-based models. Although these allow for dynamic predictions reflecting the patient's changing clinical condition, they also raise additional challenges in communicating risk trajectories to both clinical and public audiences (Figs 1C and 2). Early stakeholder engagement should ensure that the presentation of model outcomes and uncertainty supports the clinical decision-making process rather than confusing it (Figs 1B and 1D). Patient, or indeed clinician, education maybe required, particularly to ensure accurate interpretation and communication of the model outcomes (eg, where causal relationships are represented or around prediction uncertainty). There is a danger otherwise that a lack of understanding may hamper successful implementation and thus a positive impact on outcomes (Figs 1D and 2).

#### Regulatory Approval

After assessing the impact of a model, regulatory approval can be sought. Regulators across health care jurisdictions increasingly recognize that software tools with an expert function for the diagnosis, prevention, monitoring, or treatment of individual patients, including risk prediction, require regulation similar to more traditional medical devices (Figs 1D and 2).^68-70^ This has consequences for the implementation of prediction models that aim to inform patient care. Robustly determining the model's impact in clinical practice is crucial in this context (Figs 1D and 2). This should consider not only model accuracy in routine care, but also the experience of those using the model, and its effect on delivered care, outcomes, and cost-effectiveness (ideally in the context of a clinical trial),^17,71^ all of which support regulatory approvals.

As stated earlier, models are more likely to be implemented successfully if integrated in existing systems and EHRs (Figs 1D and 2D). Consideration of regulatory approval will, however, be needed at an early stage to determine if the model will be approved as part of an existing medical device/software (potentially aligning with PRO collection) or as a standalone tool.^68^ These regulations have only recently been defined or are undergoing development currently.^70,72^ Indeed, a recent scoping review of guidelines informing the development of artificial intelligence–based prediction models highlighted a dearth of relevant guidance addressing the later phases of development: software development, impact assessment, and implementation.^73^ Vigilance is required to identify ongoing regulatory requirements at all stages of model development.

### Limitations to PRO Collection and Their Impact Upon Prediction

#### Challenges to Capturing Routine PROs in Cancer Care

Despite extensive evidence of their benefits, PRO implementation within routine clinical care has been patchy and not without challenges. These have been widely considered in qualitative and implementation research.^74^ Many will have consequences for the use of PROs in prediction models, while other more general issues (such as the cost and perceived workload associated with routine PRO collection) may limit implementation entirely (Figs 2A-2C), despite examples of successful implementations with evidence of improved symptom awareness and streamlining of consultations.^36,75^

#### Reasons for Absent/Missing Data and How to Address Them

An issue particularly relevant for prediction modeling is that of absent or missing data, and crucially, its potential causes (Figs 2A and 2C). Where paper questionnaire completion comes with inherent physical limitations with delayed/missing responses and risk of human error in subsequent input to electronic systems,^76^ patient participation in electronic PRO collection depends upon digital engagement. For example, although mechanisms to reduce barriers to digital engagement have been identified,^77,78^ people who are older or who have cognitive impairment, language or literacy barriers, or limited digital access and skills, among others, are less likely to engage with electronic PROs in their routine cancer care (Fig 2C).^74,79,80^ Recent improvements notwithstanding,^81^ it is paradoxical that irrespective of the desire to inform decisions for those not routinely included in clinical trials, this structural digital exclusion may systematically exclude the same patient groups, further exacerbating existing inequalities as a result of absent data.^82^ Consequently, a review is now underway in the United Kingdom to determine the extent and impact of this on inequalities in health care.^83^

Beyond these patient-level characteristics, there are also time-varying characteristics that may diminish PRO use; crucially, in this context, qualitative studies have found that patients report feeling unable to complete PROs when feeling too unwell (Fig 2C).^74,84^ Deterioration in QOL with proximity to death is well documented,^85^ thus risking systematic exclusion of individuals with poorer prognosis and introducing bias in prediction models through missing data. Conversely, patients are unlikely to continue engaging with PROs if they do not feel that this enhances their clinical care^74^; using PROs for prediction may therefore provide an additional way to make it worthwhile for patients to complete PROs.

Mechanisms to address both person-level and time-varying challenges for PRO collection are required.^78,86,87^ These can include alternative mechanisms to collect PROs; support for patients to complete PROs inside and outside the clinic; minimizing literacy and language barriers (resulting from, eg, sensory and cognitive impairment, or reduced health literacy) through PRO development and selection; cross-cultural validation of PROs to ensure understanding and accessibility for ethnically diverse patient groups; support for clinician engagement with PROs to—in turn—encourage ongoing patient engagement; allowing proxy PRO completion if appropriate, for example, by family or carers^88-90^; identifying tools that maximize patient engagement, particularly near the end of life; minimizing data sets for model development, with clear justification for adding items; and the use of statistical approaches to address missing data (see below; Figs 2A-2C).^91^

#### PRO Selection

An extensive array of PRO measures have been developed and validated for many cancer types and populations (eg, Reinhardt et al,^92^ Engstrom et al,^93^ and Salas et al^94^). When selecting which PROs to use for routine collection, meeting the needs of clinicians and patients is of primary importance (Fig 2B). Recommendations detailing possible PRO sets have been developed by the International Consortium for Health Outcome Measurement covering some, but not all, cancer diagnoses.^95^ Avoiding the use of unvalidated PRO measures to capture the same outcome (eg, nausea/sickness, overall QOL) can help subsequent transferability of model outcomes (Fig 2B).^96^

An additional criterion for selecting PROs may be their predictive value (Fig 2A). Some PROs may be strongly predictive for outcomes in the final months of life but less so where prognosis is over a year,^41^ and there will inevitably be variation between different PRO dimensions.^37^ As with all predictors, the role of PROs within a final model should be justified based on the benefit they offer in the model (further discussed below). However, selecting those of greatest relevance to routine care may facilitate wider use of routinely collected PROs, enable integration of the model into EHR systems, reduce variation of PRO collection between health care providers, and support external validation of developed models (Figs 2A-2C). Finally, despite cognitive interviews undertaken in the development of PROs, interpretation of PRO questions may differ between patients (Fig 2B) depending on their cultural background, language skills, or other individual differences.^97,98^ This may lead to increased variation in the reported outcome between patient groups, which affects model development and validity.

### Statistical and Modeling Challenges

Existing guidelines for the development, reporting, and assessment of bias in prediction models should inform the development of any prediction model, including those incorporating PROs^17,99,100^ (Figs 1B and 2). Updates to these, recognizing aspects specific to the use of artificial intelligence in modeling—such as additional model complexity, the increased risk of overfitting to data, and subsequent lack of transparency—are in progress.^101^ There are, however, a number of specific challenges in the development of models incorporating PROs.

#### Handling Missing Data

Many of the statistical challenges of developing and implementing prediction models relate to those outlined above in the implementation of PROs, particularly resulting in missing data (Figs 2A and 2C), whether due to the absence of patient subgroups or time-varying missingness. Both of these can, to a degree, be addressed using statistical methods to complement strategies for improving data completeness. The systematic absence of PRO data from specific subgroups can be partially addressed through the use of inverse probability weighting, giving greater weight to data relating to individuals who are less likely to complete PROs (Fig 2C).^102,103^ This can enhance representativeness of models for the relevant population and aligns with an increasing recognition among regulators of the risk that models pose in introducing bias in decision making as a result of missing data and limited applicability.^99,104^ In terms of managing missingness because of, for example, deteriorating clinical condition, multiple imputation can be considered where missingness occurs at random (ie, conditional upon observed covariables, eg, when a concurrent decline in PROs and blood parameters is observed^105-107^; Fig 2A). The plausibility of underpinning assumptions and alternative methods should be assessed, as imputation where data are missing not at random will reinforce bias. Finally, when considering proxy PRO reporting (eg, by family or carers in the context of increasing frailty), understanding of the impact of this on the predictive value of PROs is required.

#### Assessing the Benefit of Alternative/Additional PRO Questions in Prediction Models

Once mechanisms to reduce and mitigate missing data have been identified, analysts should carefully consider the value offered by incorporating and thus asking additional PRO questions; excessive questions may reduce engagement,^108^ particularly near the end of life (Fig 2C).^109^ Equally, differing question structures may offer varying predictive value, for example, the three-level versus five-level EQ-5D questionnaire (with the latter providing greater detail, which may be valuable for model development; Fig 2A). Decision curve analysis can assess the comparative utility of adding further questions or the yield from additional detail.^110^ The results can then be presented to the multidisciplinary team to identify the extent to which the benefit delivered by these variables is clinically meaningful and worth pursuing through modifications to data collection methods. Conversely, if free-text answers are sought from patients, the use of qualitative analysis and natural language processing can be considered to inform model development and incorporate these responses in prognostic or predictive models.

#### Ensuring Calibration Here and Now

Models will, inevitably, reflect the populations in which they are developed and the treatments available at the time. In a rapidly changing area, such as cancer care, model predictions should be monitored for temporal drift (eg, using statistical process control methods) and recalibrated when necessary (Figs 1C and 2).^111,112^ This is increasingly recognized by regulators, and mechanisms to enable recalibration in implemented models must be planned into their design, for example, through dynamic prediction systems.^113^ Indeed, such approaches can enable calibration to institution specific outcomes, thus ensuring predictions reflect local care and outcomes.^114-116^ This can be delivered within an EHR (offering a potential benefit for the implementation of PRO-based models) and will not only improve predictive performance but may also deliver greater clinical acceptance if it is understood that the model is appropriately calibrated to reflect the local population. Caution is required, however, to avoid embedding inequalities in care and outcomes through the use of such local calibration.^117^

Beyond these changes with time and place, there is a need to capture alternative treatment approaches within developed models; the question of “but what if I have this treatment?” is highly relevant to clinicians and patients alike. As such, predictions may need to incorporate causal assumptions and counterfactual predictions, requiring careful consideration of which parameters would deliver robust predictions with minimal risk of unobserved confounding.^104,118,119^ Fully addressing confounding variables (eg, because of comorbidities) is challenging; the extent to which this can be achieved must be recognized and documented to prevent model predictions being applied to individuals for whom they are not appropriate to inform clinical decisions. This can be undertaken in parallel with wider documentation of model limitations that helps to ensure model use aligns with the population, parameters, and limitations of model development (Fig 2C). For example, where prediction is the focus, comorbidities may remain important, as they can result in reduced model performance for specific populations (eg, as a result of reduced precancer mobility).

### Strengths and Limitations

This summary and the meeting it represents include the perspectives of a broad multidisciplinary group ensuring inclusion of all disciplines relevant to developing and implementing PRO-based prediction models with a potential to improve outcomes for patients with cancer. The structured meeting ensured all key areas were discussed, while the limited group size provided opportunities for all to contribute. A larger, international group including patient and public representatives might, however, have enabled incorporation of further aspects not captured here. The meeting was funded by the EuroQoL group; however, EuroQoL had no influence over the content or outcomes of the meeting.

In conclusion, this workshop and the findings generated from it, alongside the existing literature, have shown that although PROs are increasingly used in cancer care to provide a more complete picture of a patient's symptoms and QOL, they can also be useful for enriching prediction models. Although cancer care formed the primary clinical context for the workshop discussions, we anticipate this discussion paper to also be relevant for other clinical areas. In all settings, multidisciplinary working, from the earliest stages of such model development, can help to define the decision problem, deliver broad stakeholder engagement, identify and mitigate the causes of missing PRO data, and ensure the proposed model implementation strategy aligns with the clinical pathway to be addressed and the wider health care context. Although outside of the sphere of expertise for many researchers involved in early model development and validation, this work is crucial to ensuring that the predictions produced can ultimately be used to deliver benefit to patients with cancer.
